# Implantable cardioverter-defibrillator shocks and nonsustained rapid ventricular rhythms

**DOI:** 10.1007/s12471-022-01746-z

**Published:** 2022-12-20

**Authors:** H. Witteveen, P. Stellingwerf, H. F. Groenveld

**Affiliations:** Department of Cardiology, University Medical Centre Groningen, University of Groningen, Groningen, The Netherlands

A 71-year-old man presented to the emergency department because he experienced a shock from his implantable cardioverter-defibrillator (ICD). The ICD had been implanted for secondary prevention after a cardiac arrest caused by ventricular fibrillation of unknown origin. The patient reported 3 ICD shocks in the past month, all of which had occurred during rest. The patient had remained conscious during all these events and had not experienced prodromal symptoms.

This time, he felt pain located near the ICD and therefore presented himself to the emergency department. Blood tests showed no abnormalities. An electrocardiogram (ECG) and chest radiograph were also performed (Figs. 1 and 2). What is your diagnosis?

**Fig. 1:**
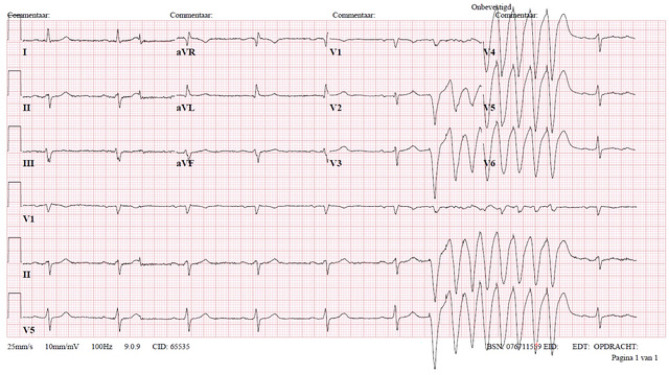
Electrocardiogram performed at the emergency department

**Fig. 2:**
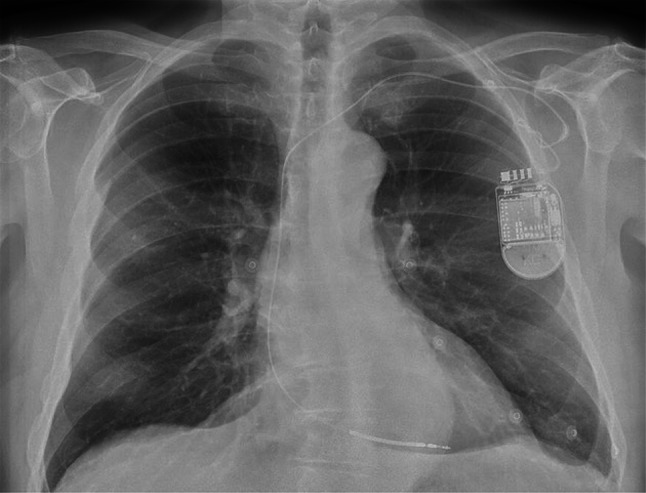
Chest radiograph taken at the emergency department

## Answer

You will find the answer elsewhere in this issue.

